# American Dental Society of Europe

**Published:** 1874-10

**Authors:** Cyrus M. Wright


### THE AMERICAN DENTAL SOCIETY OF EUROPE.

This Society held its second annual session meeting in the beautiful city of Geneva, Switzerland, on the 2d of July, 1874. The Society was called to order at 10 o'clock, a. m., in a fine saloon of the Hotel de la Paix by the President, Dr. Terry, of Zurick. A prayer was offered by the Rev. Dr. Bacon, of

America; after which an address was given by the President as the constitution requires. It was followed by an address of welcome from Dr. Field, of Geneva. The old members present were Drs. Terry, Zurick; Williams, Geneva; Van Marter, Neuchatel; Wright, Basel; Field, Geneva; and the following new members were admitted:

Drs. F. P. Abbott, S. H. Dumont and T. Paelsch, Berlin; J. B. Wasson, Rome; H. Shelly, Geneva; Jno. Crane and Chas. Kingsly, Paris; N. B. Gregory, Lyons; J. W Spear, Berne. Drs. Jenkins and Young, of Dresden, applied by telegram for membership which was laid over for one year. Other dentists from different cities in Europe appeared before the Membership Committee, but according to the constitution could not legally be admitted to active membership, as this is as far as possible an American Dental Society.

The following subjects for essays and discussions occupied the attention of the Society for three days: 1st, Pivot teeth; 2d Local peculiarities and diseases of the teeth in different points of Europe; 3d, What does experience teach to be the best material for filling teeth; 4th, Have smooth faced pluggers any advantages over serrated; 5th, Causes that underlie the decay and loss of teeth; 6th, Diseases of the antrum and treatment; 7th, Operations hurriedly made and their opposites; 8th, Difficulties and compensations of dentistry compared with other branches of the medical profession.

Essays were read by the following gentlemen:

Dr. J. G. Van Marter, on Pivot teeth.

Dr. G. W. Field, on Causes that underlie the decay and loss of the teeth.

Dr. C. M. Wright, Local peculiarities of the teeth of Europe.

Dr. N. W. Williams, on Smooth faced Pluggers.

Dr. J. B. Wasson, on Diseases of the Antrum.

Dr. F. M. Fay, on Diseases of the Antrum.

Dr. C. M. Wright, on What does experience teach to be the best material for filling teeth.

The following were elected officers for one year:

President, Dr. J. G. Van Marter, Neuchatel;

Vice-Pres. Dr. G. W. Field, Geneva.

Rec. Sec., Dr. C. M. Wright, Basel;
Cor. Sec., Dr. C. T. Terry, Zurick;
Treas., Dr. N. W. Williams, Geneva;

Increased interest was displayed by all in the new society, and the fact that the little society, originating one year ago in little Switzerland, already brings distinguished members of the profession from the great capitols of Europe, from Berlin, Rome, Paris and other cities, proves the necessity felt for an American Dental Society, and augurs well for its future. The discussions were animated and interesting, and the number of essays offered will compare favorably with any sister society in our much loved America. As it is a necessary qualification for membership that the applicant shall be a graduate of an American Dental College, and that he shall be proposed by one member and vouched for by two others, before passing the ordeal of the membership committee of three; it may be seen that this society is not so much missionary in its character as some of the societies at home; but a dental society that will confer honor on the member, and though its number may never be very large, its membership will be high and its code precise.

The next meeting will be held on the first Monday of August, 1875, in Hamburg, near Frankfort on the Main.

Cyrus M. Wright, Secretary.

---

Married at the Presbyterian Church, at Olean, N. Y., Sept. 9th, 1874, by the Rev. N. M, Clute, Dr. J. C. Oldham of Springfield, Ohio, to Miss Eva E. Osterhout, of Olean.

We hope that the joy and bliss of the happp couple, will remain and increase from this to the end of life's journey, and that all this shall constitute a mere beginning of that which is to come after.

